# A Light-Weight Practical Framework for Feces Detection and Trait Recognition

**DOI:** 10.3390/s20092644

**Published:** 2020-05-06

**Authors:** Lu Leng, Ziyuan Yang, Cheonshik Kim, Yue Zhang

**Affiliations:** 1 School of Software, Nanchang Hangkong University, Nanchang 330063, China; drluleng@gmail.com (L.L.); zhangyuecs1998@163.com (Y.Z.); 2 School of Information Engineering, Nanchang University, Nanchang 330031, China; 3 Department of Computer Engineering, Sejong University, Seoul 05006, Korea

**Keywords:** light-weight framework, feces trait recognition, object detection, visual sensor, illumination normalization method, convolutional neural network

## Abstract

Fecal trait examinations are critical in the clinical diagnosis of digestive diseases, and they can effectively reveal various aspects regarding the health of the digestive system. An automatic feces detection and trait recognition system based on a visual sensor could greatly alleviate the burden on medical inspectors and overcome many sanitation problems, such as infections. Unfortunately, the lack of digital medical images acquired with camera sensors due to patient privacy has obstructed the development of fecal examinations. In general, the computing power of an automatic fecal diagnosis machine or a mobile computer-aided diagnosis device is not always enough to run a deep network. Thus, a light-weight practical framework is proposed, which consists of three stages: illumination normalization, feces detection, and trait recognition. Illumination normalization effectively suppresses the illumination variances that degrade the recognition accuracy. Neither the shape nor the location is fixed, so shape-based and location-based object detection methods do not work well in this task. Meanwhile, this leads to a difficulty in labeling the images for training convolutional neural networks (CNN) in detection. Our segmentation scheme is free from training and labeling. The feces object is accurately detected with a well-designed threshold-based segmentation scheme on the selected color component to reduce the background disturbance. Finally, the preprocessed images are categorized into five classes with a light-weight shallow CNN, which is suitable for feces trait examinations in real hospital environments. The experiment results from our collected dataset demonstrate that our framework yields a satisfactory accuracy of 98.4%, while requiring low computational complexity and storage.

## 1. Introduction

Digestive diseases are a serious threat to human health. A quick, automatic, accurate, and robust fecal examination approach could greatly reduce the burden on medical inspectors. Unfortunately, this type of approach has remained elusive due to the scarcity of datasets and the low accuracy, and accordingly time-consuming manual examination methods are still widely used in most hospitals. In manual examinations, medical inspectors need to be close to the feces samples, which leads to a tremendous risk of cross infection. Moreover, fecal examinations are a terrible work due to the overpowering stench. Since January 2020, a lot of people have been infected by a novel coronavirus (COVID-19), and lots of medical staff have been infected owing to continuous contact with sources of infection, which not only include saliva, but also feces. Fecal examinations are highly important in the clinical diagnosis of digestive diseases. If feces samples can be acquired with vision sensors, and the feces in the acquired images can be quickly, automatically, accurately, and robustly detected and recognized, medical staff will be able to save much diagnosing time and patients will be able to receive their diagnostic reports quickly. Fecal examinations are widely applied to evaluate the probability of getting or relapsing into digestive diseases [1], so various related methods have been proposed in recent years.

Fecal examinations can be briefly divided into macroscopic examinations and the microscopic examinations. The camera sensor-based macroscopic examination is a fast and convenient assessment method for prescreening various terrible digestive diseases. A trait examination is an important item in fecal macroscopic examinations, so we proposed a light-weight practical framework for trait recognition, which works well in a real hospital environment. The model should be updated with the increasing number of collected samples; therefore, the subsequent model update is also considered in this paper. Because of the light-weight structure, our framework can be fine-tuned with low hardware requirements. Moreover, the light-weight framework can be conveniently embedded into the mobile fecal examination machine.

**Figure 1:**
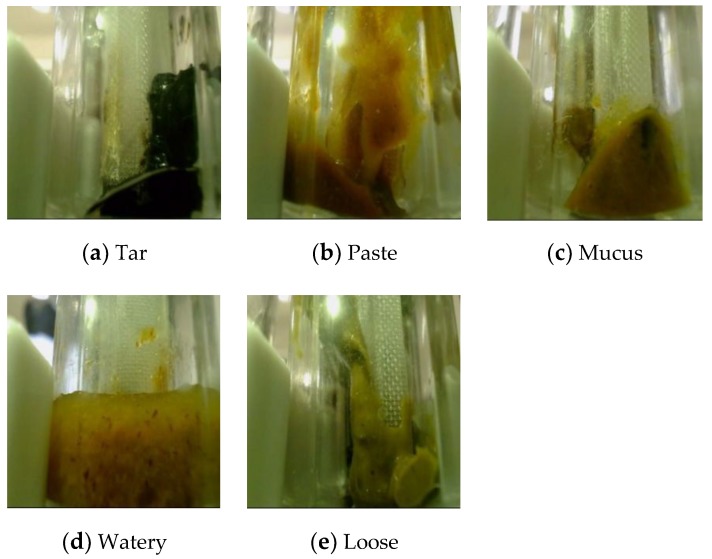
Feces trait classes.

Because of the scarcity of fecal trait datasets labeled by professional doctors, few related automatic diagnosis systems have been designed and this research field is developing slowly. To our best knowledge, the only report on fecal trait recognition is found in [2]. However, this method cannot maintain its recognition performance in real hospital environments because the dataset was collected in uncontrollable environments. In order to alleviate this problem, we tried to compile a dataset in which all of the acquired images are only used for scientific research with the consent of the patients. It is worth emphasizing that all the images were labeled by professional doctors, so the dataset has a high research and medical value. Moreover, the fecal images were carefully classified into five classes, as shown in Figure 1, namely, tar, paste, mucus, watery, and loose.

Our approach is composed of three stages: illumination normalization, object detection, and classification diagnosis. The first stage and the second stage are combined into the preprocessing stage. The illumination condition contains the illumination source and the illumination level. The illumination sources in our fecal detection machine are astigmatism, such as natural light and lamplights that are unlike spotlights, so the principal illumination is the variance in the illumination level. Since the illumination conditions are not always exactly uniform in different hospitals, the digital images acquired with the camera sensors are not always at the same illumination level, so the images need to be normalized to a uniform illumination level. Then, the feces object is detected with segmentation to remove the disturbance of the background. Finally, a light-weight practical convolutional neural network (CNN) is proposed for the feature extraction and trait recognition. The main contributions of this paper include:

1. A novel research field for public health is proposed, in which the fecal image dataset is collected in a real hospital environment and labeled by professional doctors. This dataset has a high clinical value and a high medical value;
2. A quick automatic accurate and robust diagnosis framework is proposed. The feces object is accurately detected with a well-designed threshold-based segmentation scheme on the selected color component to reduce the background disturbance. We find that the CNN does not resist the illumination variance well. In contrast, our framework has a strong tolerance for illumination variances, and the trait classification accuracy is satisfactory;
3. Our light-weight framework is economical and meets the requirements of practical applications. The computational complexity is low and the number of parameters is small. It is feasible and convenient to fine-tune the structure with a common hardware source.

The rest of this paper is organized as follows. Section 2 introduces the related work. Section 3 specifies our framework in detail. The experiments and discussion are in Section 4. The conclusions are drawn in Section 5.

## 2. Related Works

Fecal examinations can be roughly divided into traditional methods and computer-aided methods. Traditional fecal examinations include physical-based and chemical-based methods. Kopylov et al. [3] confirmed the correlation between calprotectin and the small bowel, and assessed the small bowel diagnostic accuracy based on calprotectin. Costea et al. [4] tested 21 representative DNA extraction protocols and recommended a standardized fecal DNA extraction method. Teimoori et al. [5] applied a developed recombinant O. viverrini cathepsin F to diagnose human opisthorchiasis. Inpankaew et al. [6] compared Kato–Katz and a simple sodium nitrate flotation technique in the identification of eggs in feces samples. Cai et al. [7] applied a TaqMan based real-time polymerase chain reaction to detect C. sinensis DNA in the feces sample. The methods mentioned above require lots of demanding professional skills, expensive sensors, instruments, and reagents. Moreover, these methods may cause pollution in environment [8].

Computer-aided medical diagnosis systems for macroscopic examinations have several advantages, such as the quick examination speed, high accuracy, low risk of cross infection, and low levels of professional skills required. Many automatic diagnosis systems are designed with computer technology. Theriot et al. [9] used a logistic model to classify the patients with non-C. difficile diarrhea, C. difficile infection, and the patients who are asymptomatically colonized with C. difficile. Carvalho et al. [10] combined fuzzy logic and a support vector machine (SVM) to diagnose lung nodules; the fuzzy rule was designed by a professional doctor. Similarly, Soundararajan et al. [11] proposed a fuzzy logic-based knowledge system for tuberculosis recognition. The aforementioned methods are based on manual feature extraction.

With the rapid development of artificial intelligence, especially deep learning, in recent years, convolutional neural networks (CNN) have been widely studied and have achieved excellent results in different tasks related to computer vision, such as image enhancement [12], segmentation [13], tracking [14], detection [15], and recognition [16,17,18]. The features learned with the CNN do not heavily rely on manual modeling, so their robustness and accuracy are usually better than for manual methods. In the intelligent healthcare field, CNNs do well in analyzing medical images [19]. Sun et al. [20] implemented three network structures and some traditional methods, and their deep belief network yielded a satisfactory accuracy in diagnosing lung cancer based on computed tomography (CT) images. Arabasadi et al. [21] proposed a hybrid method in which a genetic algorithm was used to initialize the parameters, and the CNN was used to extract the features and classify cardiovascular diseases. Oktay et al. [22] incorporated prior anatomical knowledge into CNNs through a novel regularization model. Since fusion can improve the performance in many ways [23,24,25], Liu et al. [26] proposed a novel network layer that effectively fuses the global information from the input, and a novel multi-scale input strategy that acquires multi-scale features. Li et al. [27] proposed a novel 3D self-attention CNN for the low-dose CT denoising problem; the structure acquired more spatial information. Tschandl et al. [28] trained two CNNs with dermoscopic images and clinical close-ups images, respectively, and combined the outputs of CNNs to diagnose nonpigmented skin cancer. In addition, Singhal et al. [29] applied a CNN to analyze the emotion variances of people based on electroencephalograms.

Object detection is widely used in the medical diagnosis field. Many object detection methods have been proposed and have yielded remarkable results in recent years [30,31]. Yang et al. [32] proposed a novel object detection method that combined multi-scale features and an attention-based rotation network. Pang et al. [33] improved “you only look once” (YOLO) [34] to detect concealed objects. Yang et al. [35] proposed a real-time cascaded framework to detect tiny faces. Yuan et al. [36] proposed a scale-adaptive CNN to detect occluded targets and track them. Zhao et al. [37] detected the salient object according to the difference between the feature maps of different depths. Fu et al. [38] proposed a general unified framework to detect the salient object, which is composed of “skip-layer” architecture, “top-down” architecture, “short-connection” architecture, and so on. These CNN-based methods yield satisfactory accuracies, but they require a large amount of data and high-performance hardware to train the networks. Because of the particularity of feces samples, neither the location nor the shape is fixed, it is unfeasible to directly label the images to train CNNs for feces object detection.

Vision systems can generate more efficient and diverse information than the other sensory organs [39,40,41], so medical images are usually acquired by visual sensors. However, images typically contain much private information, and few patients are willing to provide their pathological samples, certainly including fecal samples. Few researchers have investigated computer-aided fecal examinations because of the lack of datasets. Hachuel et al. [2] applied ResNet [42] to classify feces macroscopic images into three classes, namely, constipation, normal, and loose. The images were collected in uncontrollable environments. The structure is very deep, and it is unsuitable for a real hospital environment, which is a unified and controllable environment. Nkamgang et al. [43] extracted histogram orientation gradient (HOG) features from the microscopic images and applied a neuro-fuzzy classifier for the diagnosis of intestinal parasite diseases. Yang et al. [44] proposed a shallow CNN dubbed StoolNet to classify the colors of fecal images.

## 3. Materials and Methods

### 3.1. Overview of the Framework

**Figure 2:**
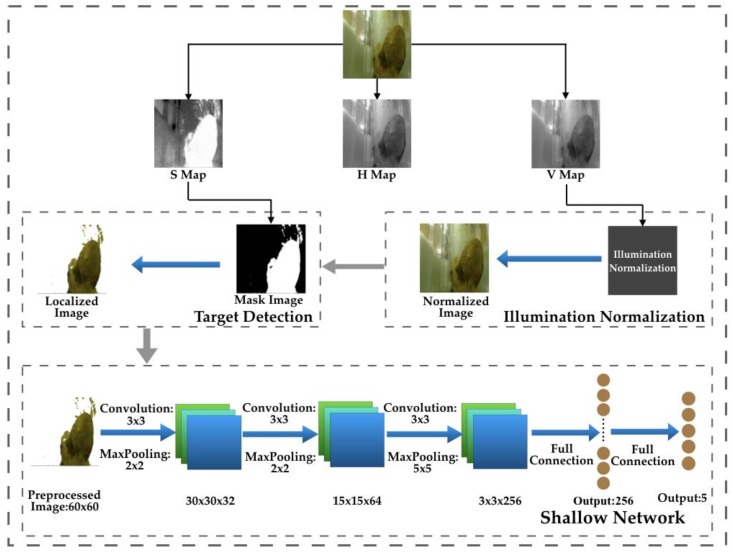
The framework of fecal trait examination.

Routine fecal examinations are important to the patients with digestive diseases, and trait examination results can be used to diagnose various related diseases. In this paper, we propose a quick, automatic, accurate, and robust framework for fecal trait examinations, which consists of three stages, as shown in Figure 2. The input images should be transformed to hue, saturation, and value (HSV) color space, and the illumination normalization and target detection are conducted on the V map and H map, respectively. Illumination variance deteriorates the classification accuracy. In real environments, digital images are acquired with vision sensors; however, the illumination level is not always fixed. Hence, a simple but efficient illumination normalization method is necessary to improve the robustness. The feces object is accurately detected with a well-designed threshold-based segmentation scheme on the selected color component to reduce background disturbance. Finally, the preprocessed images are input to a shadow CNN that is suitable for the fecal trait classification task.

### 3.2. Illumination Normalization

A good illumination tolerance is very important for feature extraction [45,46]; the requirement of the robustness against the illumination variance is very high, especially in the medical diagnosis field. In order to reduce the computational complexity and improve the robustness, a simple but effective illumination normalization method is proposed.

Red, green, and blue (RGB) is a typical image color space. However, the three channels are all sensitive to illumination variance. Therefore, RGB color space should be transformed to HSV color space. In HSV color space, only the value channel is sensitive to illumination variance. Therefore, the images are transformed into HSV color space first. The value range of H is [0, 360]. The value of H is calculated as

(1) $$h=\{\begin{array}{l} 0{}^{\circ},\;if\;\;max=min\\ 60{}^{\circ}\times \frac{g-b}{max-min}+0{}^{\circ},\;if\;\;max=r\;and\;g\geq b\\ 60{}^{\circ}\times \frac{g-b}{max-min}+360{}^{\circ},\;if\;max=\;r\;and\;g<b\\ 60{}^{\circ}\times \frac{b-r}{max-min}+120{}^{\circ},\;\;if\;max=g\\ 60{}^{\circ}\times \frac{r-g}{max-min}+240{}^{\circ},\;\;if\;max=b \end{array}$$

where r, g, and b are the values in the red, green, and blue channels, respectively; max=max(r,g,b), min=min(r,g,b), max and min are the functions to select the maximum value and the minimum value from the inputs; and h is the hue value.

The value of S is calculated as

(2) $$=\{\begin{array}{c} 0,\;\;\;if\;max=0\\ \frac{max-min}{max},\;\;otherwise \end{array}$$

where s is the saturation value.

The value of V is the maximum value of r, g, and b, which is repressed as

(3) v=max

where v is the value in the value channel.

Commonly, if the training set contains the images under various illuminations, the recognition accuracy will be good when the illuminations of the testing images are the same as those of the training set, which will be confirmed in Section 4. However, in practical environments, it is difficult to collect images under different illuminations. It is challenging to test the images at different illumination levels while the training images are at a single illumination level. Since the backgrounds of the images acquired with our machine are approximately uniform, we calculate the standard illumination value, i.e., the average illumination value of the background pixels of all training images. The standard illumination value is used to adjust the illuminations of the testing images. At first, the reference number is calculated as

(4) $$v_{s}=\frac{1}{n}\sum_{i=1}^{n}v_{i}$$

where n is the number of training images, $v_{i}$ is the average illumination value of the background pixels of the i-th training image, and $v_{s}$ is the standard illumination value.

The values of the three channels are calculated according to the reference number, because the three channels are changed by the equal reference value; the differences of the hue and the texture between the original image and the referenced image are not huge. The values after illumination normalization are calculated as

(5) $$R_{o}=R\times \frac{V_{t}}{V_{s}}$$

where R and $R_{o}$ are the original and normalized red channels, respectively, and $v_{t}$ is the average illumination value of the background pixels of the testing image.

(6) $$G_{o}=G\times \frac{V_{t}}{V_{s}}$$

(7) $$B_{o}=B\times \frac{V_{t}}{V_{s}}$$

Similarly, the green and blue channels are processed in the same way.

### 3.3. Object Detection

**Figure 3:**
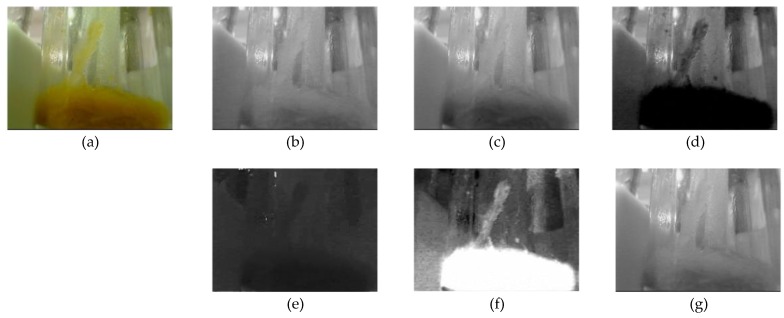
The grayscale maps in different color channels. (**a**) Represents the original image; (**b–g**) represent the red, green, blue, hue, saturation, and value channels, respectively.

The background could decrease the recognition accuracy. In order to suppress the background disturbance and converge it into a short epoch, the object regions are automatically detected with an unsupervised threshold segmentation method that yields a satisfactory detection accuracy. The threshold-based method typically works on a grayscale map. Compared with other color components, saturation is a good feature for feces object detection, which is shown in Figure 3.

**Figure 4:**
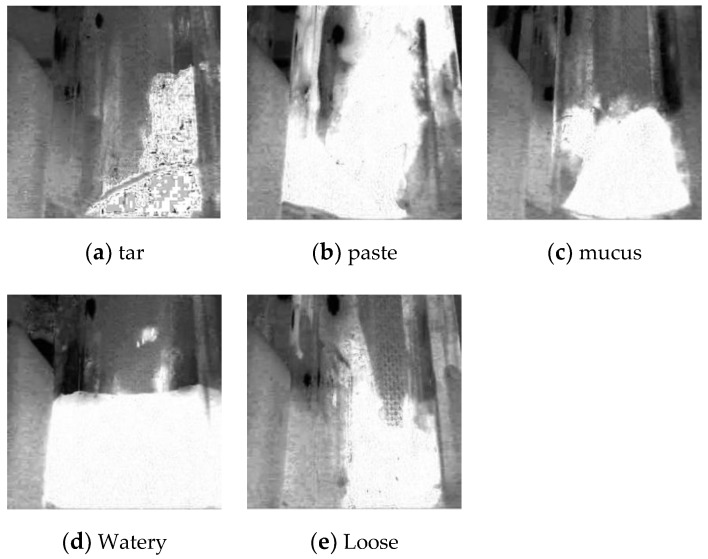
Saturation maps of different classes.

The saturation maps of five different classes are shown in Figure 4. The saturation channel can always yield a good distinguish performance.

The automatic detection method is a threshold-based method on the grayscale map, with which an adaptive threshold is computed. The proportions of the foreground and the background pixels are first calculated.

(8) $$\{\begin{array}{c} w_{0}=\frac{N_{0}}{M\times N}\\ w_{1}=\frac{N_{1}}{M\times N} \end{array},\;w_{0}+w_{1}=1\;and\;N_{0}+N_{1}=M\times N$$

where $w_{0}$ and $w_{1}$ are the proportions of the foreground and the background pixels, respectively; $N_{0}$ and $N_{1}$ are the numbers of foreground and background pixels, respectively; and M×N is the size of the image.

The average grayscale value of the image is calculated as

(9) $$\mu =w_{0}\times \mu_{0}+w_{1}\times \mu_{1}$$

where μ, $\mu_{0},$ and $\mu_{1}$ are the average grayscale value of the whole image, the foreground pixels, and the background pixels, respectively.

Finally, the greatest difference between the grayscale values of the background and the foreground yields a satisfactory segmentation effect. Meanwhile, the grayscale value is considered as the segmentation threshold.

(10) $$g=w_{0}\times (\mu_{0}-\mu {)}^{2}+w_{1}\times (\mu_{1}-\mu {)}^{2}$$

where g is the inter-variance between the foreground and background. The objective is to search the adapted threshold to maximize g.

### 3.4. Shallow CNN

In this paper, a shallow CNN is proposed to recognize fecal traits, the network structure is shown in Figure 2. The input of the network is resized as 60 × 60. The first three layers are all convolutional layers with a kernel size of 3 × 3, and the numbers of filters are 32, 64, and 256, respectively. The convoluted results are all passed to the max pooling layers. The two full connection layers are with a 256-dimensional vector and five-dimensional vector, respectively. In order to conveniently reproduce the structure, the key functions are defined as follows:

ReLU is the activation function:

(11) f(x)=max(0,x)

Dropout [47] is applied on the full connection layers to avoid the overfitting problem. The outputs of the network are normalized using the softmax function:

(12) $$softmax\left(y_{i}\right)=\frac{e^{y_{i}}}{\sum_{j=1}^{m}e^{y_{j}}}$$

where i is the class index, $y_{i}$ is the probability of i-th class, and m is the number of classes.

Adam optimization [48] is applied to update parameters, and the loss function is a logistic function:

(13) $$L\left(p,q\right)=-\sum_{i=1}^{n}p_{i}^{T}\cdot log(q_{i})$$

where $p_{i}^{T}$ is the ground truth probability of i-th sample, $q_{i}$ is the predicted probability, and n is the number of samples.

## 4. Experiments and Discussion

The experimental setup is as follows: Intel Core i5-8250U CPU, 8GB internal storage, NVIDIA GeForce mx150 GPU. All codes were written in Python. The Python compiler is Pycharm. The deep learning framework is Tensorflow [49].

**Figure 5:**
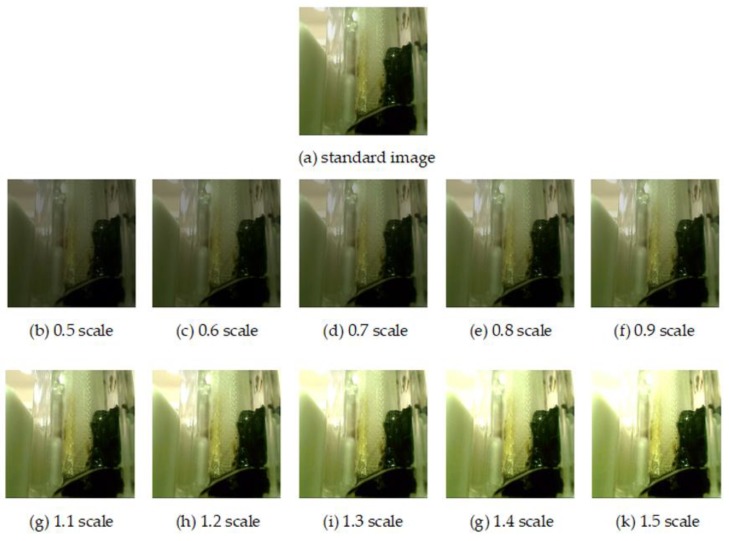
Feces images under different illuminations.

As far as we know, there is no public fecal examination dataset until now. Even though fecal examinations are very important to healthcare, the lack of a dataset has suppressed the development of the related studies. Professional doctors carefully categorized the fecal images into five typical trait classes: tar, paste, mucus, watery, and loose. Illumination variance typically deteriorates the recognition accuracy, so we needed to test our approach under different illumination conditions. However, it is difficult to collect the fecal images under different illuminations, so we simulated the effects of various illumination scales, as shown in Figure 5. We collected and augmented the fecal trait images using the method in [50]. Each image was rotated three times and inverted. If the shape of the object in one of the newly augmented image existed in the previous augmented images, this augmented image was deleted. Hence, each original image has seven augmented images. The images after augmentation were salted with Gaussian noise. Finally, the total number of images is 6336.

In all experiments, 75% and 25% of the dataset were set as the training set and the testing set, respectively. In order to suppress the information learned from the shape, the images with the same shape were put only in the training set or in the testing set. The shape of an original image was the reference shape, its rotation and inversion generated different shapes, while the shapes of the other augmented images were considered to have the same shape as the reference shape. Two main reasons motivated us to do this: first, in order to verify the effect of our method in a tough environment, we deliberately increased the difficulty by reducing the number of shape styles that could be learned in training set; second, it is rare to find two feces samples with the same shape in practice, so this operation is reasonable.

**Figure 6:**
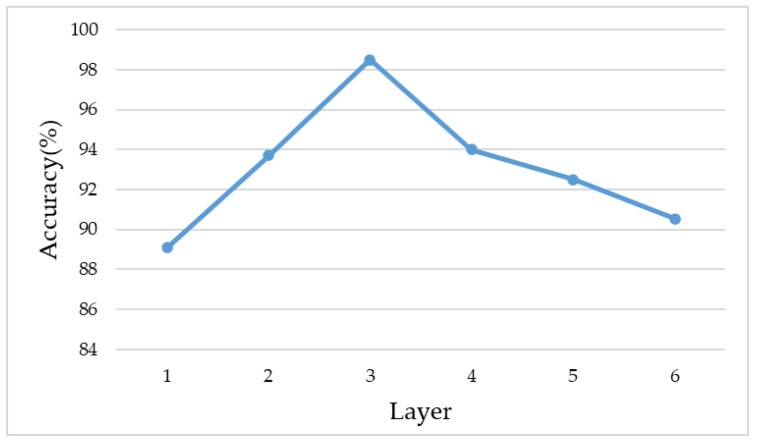
Accuracies with various layer numbers.

All images were preprocessed, the preprocessing stage includes illumination normalization and object detection with segmentation. The relationship between the network depth and its recognition accuracy is shown in Figure 6. For the trait recognition task, three layers is a better choice than the other structures. When the number of layers was less than three, the corresponding layers were removed. When the number of layers was larger than three, the number of kernels was doubled layer by layer, and the step size remained one.

**Figure 7:**
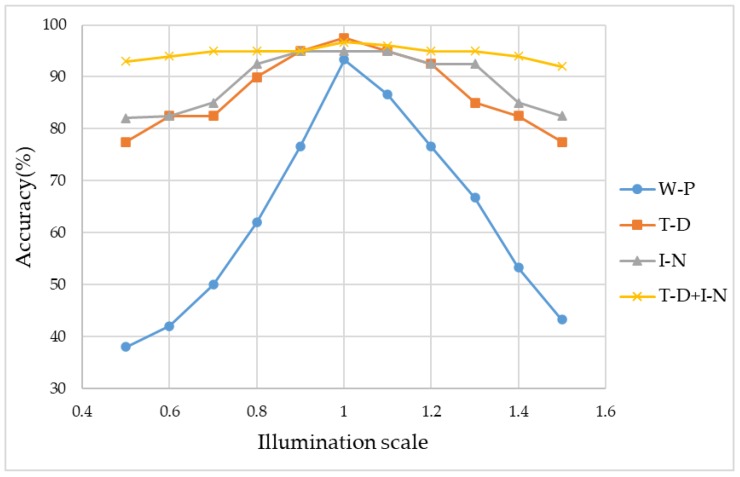
Accuracies at different illumination scales.

Images acquired with vision sensors in different hospitals are not always at the same illumination level, so we tested the illumination variance tolerance of our approach. If the training images and testing images were under highly different illumination levels, the recognition accuracy could be terrible. Thus, in this experiment, the training set consisted of images under a single illumination, while the testing set consisted of images with different illuminations. As shown in Table 1, illumination normalization and object detection can improve the robustness against illumination variance. Moreover, recognition accuracy is always the best when both the two preprocessed methods are jointly applied. As shown in Figure 7, “W-P”, “T-D” and “I-N” denotes “without preprocessing”, “target detection”, and “illumination normalization”.

The target detection method is insensitive to illumination variance, because the proportion between the foreground and the background does not depend on illumination variance. The normalized Hamming distance measures the dissimilarity between the segmented images under the standard illumination and other illuminations. According to Table 1, all the normalized Hamming distances are very low, which confirms that our target detection method is insensitive to illumination variance.

There are very few fecal trait recognition methods, so we compared our method with the method in [2] and the related state of the art [44]. In [2], the method was not designed for a real hospital environment. Furthermore, there are several substantial differences between this paper and [44], which are summarized as follows:

1. The recognition tasks are different. In [44], the method is designed for fecal color recognition, but the main objective of this paper was to design a quick, automatic, accurate, and robust method to classify the traits of fecal images. Color recognition and trait recognition are both important for macroscopic examinations, but typically trait recognition is more difficult than color recognition;
2. The method described in [44] cannot maintain its level of performance in the task presented in this paper, which is demonstrated by the experimental results. The developed novel method in this paper can work well in the trait classification task;
3. The method described in [44] cannot work well at different illumination levels. In contrast, the illumination problem has been solved well in this paper.

According to Table 2, when the training set was composed of the images under different illuminations, the accuracy of our method was at least 7.78% higher than the other methods. Besides, when the training set was composed of the images under single illumination, our method yields at least a 13.96% higher accuracy than the other methods. The method described in [2] was designed for uncontrollable environments, which means that the structure is too bloated to converge in controllable environments. In addition, the number of classes in our dataset is more than in their dataset, which implies that the classification difficulty was higher for our dataset in the task. It can be concluded that a deeper depth and a bigger number of parameters do not always yield a better recognition accuracy; however, they would lead to difficulties converging, especially in controllable environments. The depth of the CNN in [2] is 18, while the depth of our network is only three. Hence, our method can converge fast and yields a high recognition accuracy in a real hospital environment.

## 5. Conclusions

In this paper, we propose a novel, quick, automatic, accurate, and robust fecal trait examination approach. A valuable fecal trait dataset was collected in a real hospital environment and all the images were labeled by professional doctors. The feces object was accurately detected with a well-designed threshold-based segmentation scheme on the selected color component to reduce the background disturbance. In addition, the illumination normalization scheme has a strong tolerance for illumination variance, and the recognition accuracy meets the business requirements. As a result of the light-weight structure, the computational complexity and the storage cost are both low, which is necessary for the application of automatic fecal examination machines and some edge devices, such as hand-held examination devices. Meanwhile, the shallow structure means it is feasible and convenient to fine-tune when more samples are collected. In the future work, we will try to develop a general fecal examination system with more functions and collect more samples to enlarge our dataset.

## Figures and Tables

**Table 1:** Normalized Hamming distances between the segmented images under different illuminations.

Illumination Scale	Tar	Paste	Mucus	Watery	Loose
0.5	0.0033	0.0047	0.0046	0.0053	0.0045
0.6	0.0028	0.0047	0.0039	0.0039	0.0039
0.7	0.0025	0.0038	0.0034	0.0029	0.0037
0.8	0.0022	0.0020	0.0034	0.0029	0.0028
0.9	0.0026	0.0024	0.0033	0.0021	0.0025
1.1	0.0058	0.0038	0.0041	0.0042	0.0060
1.2	0.0118	0.0056	0.0100	0.0060	0.0105
1.3	0.0281	0.0165	0.0128	0.0145	0.0191
1.4	0.0350	0.0282	0.0239	0.0203	0.0235
1.5	0.0417	0.0486	0.0380	0.0379	0.0282

**Table 2:** Accuracies of different methods.

Training Set	ResNet [2]	StoolNet [44]	Proposed
Single illumination	43.01%	84.37%	98.33%
All illumination	43.74%	90.62%	98.40%
